# Association between maternal heavy metal exposure and Kawasaki Disease, the Japan Environment and Children’s Study (JECS)

**DOI:** 10.1038/s41598-024-60830-z

**Published:** 2024-04-30

**Authors:** Takanori Yanai, Satomi Yoshida, Masato Takeuchi, Chihiro Kawakami, Koji Kawakami, Shuichi Ito, the Japan Environment and Children’s Study Group, Michihiro Kamijima, Shin Yamazaki, Yukihiro Ohya, Reiko Kishi, Nobuo Yaegashi, Chisato Mori, Zentaro Yamagata, Hidekuni Inadera, Takeo Nakayama, Tomotaka Sobue, Masayuki Shima, Hiroshige Nakamura, Narufumi Suganuma, Koichi Kusuhara, Takahiko Katoh

**Affiliations:** 1 Department of Pharmacoepidemiology, Kyoto University Graduate School of Medicine and Public Health, Yoshida-Konoecho, Sakyo-ku, Kyoto, 606-8501 Japan; 2 Kanagawa Regional Center for JECS, Yokohama City University Graduate School of Medicine, 3-9 Fuku-ura, Kanazawa-ku, Yokohama, 236-0004 Japan; 3 Department of Pediatrics, Yokohama City University Graduate School of Medicine, 3-9 Fuku-ura, Kanazawa-ku, Yokohama, 236-0004 Japan; 4 Nagoya City University, Nagoya, Japan; 5 National Institute for Environmental Studies, Tsukuba, Japan; 6 National Center for Child Health and Development, Tokyo, Japan; 7 Hokkaido University, Sapporo, Japan; 8 Tohoku University, Sendai, Japan; 9 Chiba University, Chiba, Japan; 10 University of Yamanashi, Chuo, Japan; 11 University of Toyama, Toyama, Japan; 12 Kyoto University, Kyoto, Japan; 13 Osaka University, Suita, Japan; 14 Hyogo Medical University, Nishinomiya, Japan; 15 Tottori University, Yonago, Japan; 16 Kochi University, Nankoku, Japan; 17 University of Occupational and Environmental Health, Kitakyushu, Japan; 18 Kumamoto University, Kumamoto, Japan

**Keywords:** Epidemiology, Paediatric research, Environmental impact

## Abstract

Kawasaki disease (KD) is an acute systemic vasculitis primarily affecting young children, with an unclear etiology. We investigated the link between maternal heavy metal exposure and KD incidence in children using the Japan Environment and Children’s Study, a large-scale nationwide prospective cohort with approximately 100,000 mother–child pairs. Maternal blood samples collected during the second/third trimester were analyzed for heavy metals [mercury (Hg), cadmium (Cd), lead (Pb), selenium (Se), manganese (Mn)], divided into four quartiles based on concentration levels. KD incidence within the first year of life was tracked via questionnaire. Among 85,378 mother–child pairs, 316 children (0.37%) under one year were diagnosed with KD. Compared with the lowest concentration group (Q1), the highest (Q4) showed odds ratios (95% confidence interval) for Hg, 1.29 (0.82–2.03); Cd, 0.99 (0.63–1.58); Pb, 0.84 (0.52–1.34); Se, 1.17 (0.70–1.94); Mn, 0.70 (0.44–1.11), indicating no concentration-dependent increase. Sensitivity analyses with logarithmic transformation and extended outcomes up to age 3 yielded similar results. No significant association was found between maternal heavy metal levels and KD incidence, suggesting that heavy metal exposure does not increase KD risk.

## Introduction

Kawasaki disease (KD) is an acute systemic vasculitis that mainly affects young children^1^. Eighty-five percent of KD cases occur at age 5 years or younger, with the most common age group 6–11 months^2^. The annual KD prevalence was 18.1, 8.4, 82.7, and 264.8 per 100,000 children in the United States (US), the United Kingdom (UK), Taiwan, and Japan, respectively, from the 2020 report^3^.

The etiology of KD remains unknown, and a causal relationship between KD and its risk factors has not been established, although infectious diseases, environmental factors, and genetic susceptibility have been cited^1^. One suggested environmental factor is exposure to heavy metals during pregnancy or children themselves^4^. However, studies on the association between heavy metals and KD are based on few case reports^5,6^.

The Japan Environment and Children's Study (JECS), funded by the Japanese Ministry of the Environment, is an ongoing large birth cohort examining the impact of environmental exposure, including heavy metals, on maternal and child health^7^. The cohort measures and collects maternal blood heavy metal levels for mercury, cadmium, lead, selenium, and manganese. Since these heavy metals are transferred to the fetus^8,9^, and infants have limited variation in oral intake, the influence of the mother's prenatal environment is considered significant.

Given the above background, the present study examines the association between maternal heavy metal concentrations and the incidence of KD in infancy. A similar large-scale study has never been performed before, this is the first report of investigating maternal heavy metal and KD.

## Results

**Figure 1:**
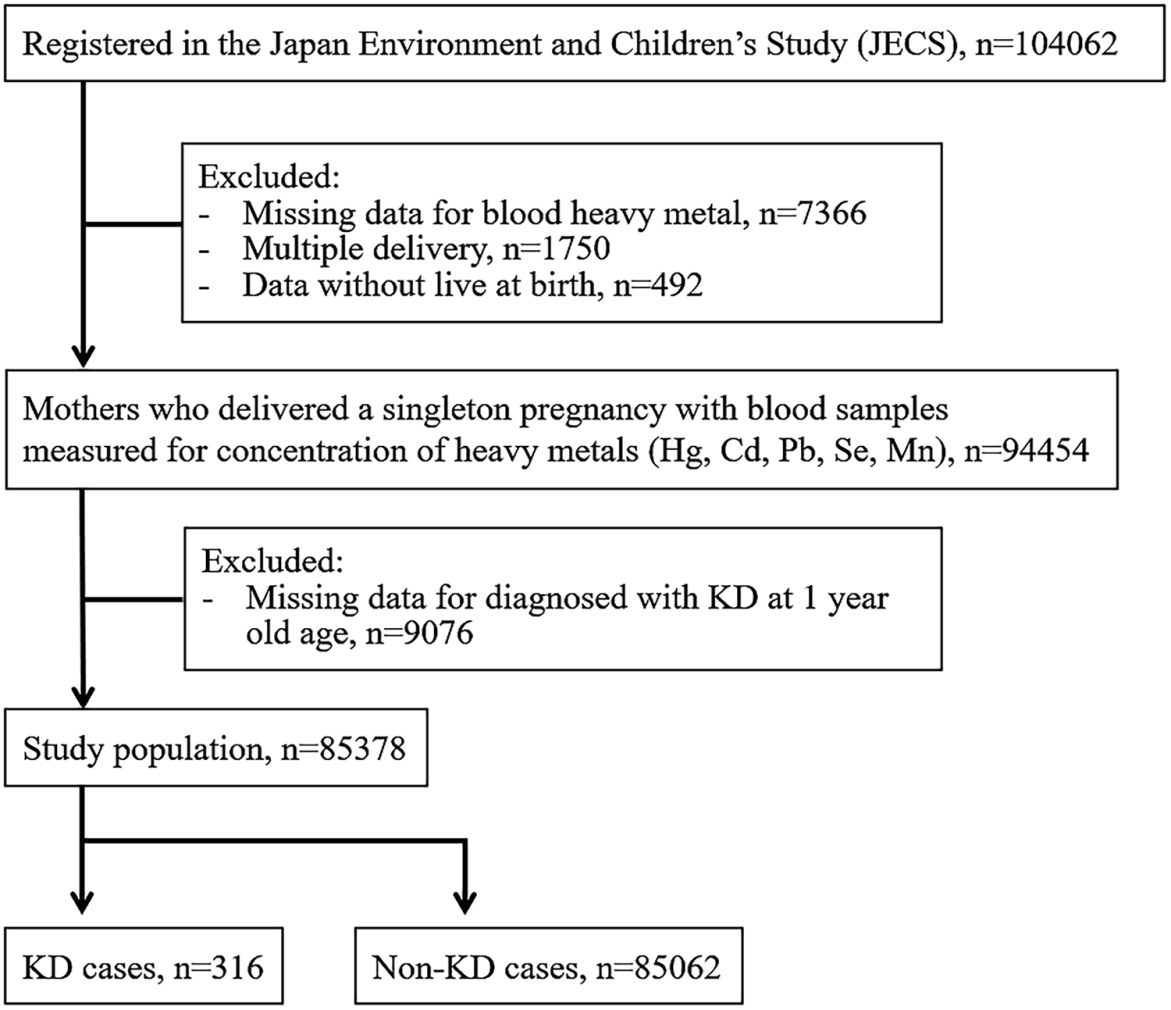
Participant flowchart.

The JECS contains records of 104,062 fetal records (Fig. 1). After excluding cases with missing data, 85,378 pairs were included in the analysis. The median maternal blood heavy metal concentrations (IQR) were 3.65 (2.55–5.20), 0.66 (0.50–0.90), 5.84 (4.69–7.31), 168 (156–182), and 15.3 (12.6–18.6) ng/g for mercury, cadmium, lead, selenium, and manganese, respectively. Among the eligible children, 51% were boys and 49% girls, the median gestational week was 39 (38–40) weeks, and birth weight was 3030 (2786–3284) g. Of these, 316 were diagnosed with KD at C1Y. The KD incidence was 3.70 per 1000 live births.

Table 1 compares the two groups: a KD group who had KD at C1Y and a non-KD group who did not have KD at C1Y. At baseline, the sex ratio, number of weeks of gestation, birth weight, domestic area, and annual household income were not significantly different between these two groups. The maternal blood heavy metal concentrations were not significantly different for mercury, cadmium, lead, selenium, and manganese by the Mann–Whitney U test. In addition, there were no significant differences between the two groups in terms of maternal history of allergy and anti-allergy medication during pregnancy. Regarding the child factors, no significant differences were observed between the two groups for feeding, acute infections, and allergic diseases.

**Table 1:** Comparison of patient characteristics.

	KD cases (N = 316)	Non-KD cases (N = 85,062)	*p*
Sex			
Male (%)	170 (53.8)	43,617 (51.3)	0.398
Female (%)	146 (46.2)	41,443 (48.7)	
Gestational week [IQR]	39 [38–40]	39 [38–40]	
Birth weight (g) [IQR]	3030 [2802–3274]	3030 [2786–3284]	0.917
Area			
Hokkaido (%)	24 (7.6)	6574 (7.7)	
Tohoku (%)	72 (22.8)	18,789 (22.1)	
Kanto (%)	56 (17.7)	16,064 (18.9)	
Chubu (%)	40 (12.7)	9606 (11.3)	
Kinki (%)	59 (18.7)	14,373 (16.9)	
Chugoku (%)	11 (3.5)	2601 (3.1)	
Shikoku (%)	15 (4.7)	5789 (6.8)	
Kyushu-Okinawa (%)	39 (12.3)	11,266 (13.2)	
(Maternal factors) Maternal blood heavy metal concentrations			
Mercury (ng/g) [IQR]	3.62 [2.63–5.34]	3.65 [2.55–5.20]	0.632
Cadmium (ng/g) [IQR]	0.67 [0.51–0.89]	0.66 [0.50–0.90]	0.320
Lead (ng/g) [IQR]	5.69 [4.79–7.07]	5.84 [4.69–7.31]	0.477
Selenium (ng/g) [IQR]	169 [158–182]	168 [156–182]	0.357
Manganese (ng/g) [IQR]	15.0 [12.8–18.3]	15.3 [12.6–18.6]	0.950
Mother's use of antiallergic medication during pregnancy			
Yes (%)	2 (0.6)	1373 (1.6)	0.256
No (%)	314 (99.4)	83,689 (98.4)	
Mother’s allergy history			
Yes (%)	166 (52.5)	46,748 (55.0)	
No (%)	150 (47.5)	38,314 (45.0)	
(Children’s factors) Abnormality at birth			
Yes (%)	22 (7.1)	5048 (6.1)	0.403
No (%)	287 (92.9)	78,387 (93.9)	
Complete breastfeeding			
Yes (%)	158 (50.2)	45,681 (53.9)	0.193
No (%)	157 (49.8)	38,995 (46.1)	
Mixed breastfeeding			
Yes (%)	139 (44.1)	33,880 (40.0)	0.150
No (%)	176 (55.9)	50,796 (60.0)	
Complete formula feeding			
Yes (%)	12 (3.8)	3269 (3.9)	1.0
No (%)	303 (96.2)	81,407 (96.1)	
Child’s history of frequent exposure to cold (over 4 times)			
Yes (%)	59 (18.7)	19,376 (22.8)	0.093
No (%)	257 (81.3)	65,686 (77.2)	
Child’s history of gastroenteritis			
Yes (%)	33 (10.4)	7898 (9.3)	0.496
No (%)	283 (89.6)	77,164 (90.7)	
Child’s history of atopic dermatitis			
Yes (%)	12 (3.8)	3640 (4.3)	0.781
No (%)	304 (96.2)	81,422 (95.7)	
Child’s history of food allergy			
Yes (%)	22 (7.0)	5590 (6.6)	0.733
No (%)	294 (93.0)	79,432 (93.4)	
Child’s history of bronchial asthma			
Yes (%)	8 (2.5)	2167 (2.5)	1.0
No (%)	308 (97.5)	82,895 (97.5)	
(Familial, socioeconomic factors) Siblings			
Yes (%)	207 (65.9)	47,626 (56.7)	0.001
No (%)	107 (34.1)	36,297 (34.3)	
Family income			
Over 8 million JPY/yr (%)	30 (9.4)	8592 (10.1)	1.0
Under 8 million JPY/yr (%)	264 (90.6)	70,342 (89.9)	

Table 2 shows the ORs of KD for each quartile of heavy metal concentrations (Q1–Q4). For mercury, 316 cases in the KD group were evenly distributed in Q1–Q4, and there was no increase in each concentration group. The adjusted ORs were 1.13 (0.71–1.80), 1.21 (0.77–1.93), and 1.29 (0.82–2.03) for Q2, Q3, and Q4, respectively, with no increase. This trend was also observed for cadmium, lead, selenium, and manganese.

**Table 2:** Odds ratios (OR) between quartile concentrations of maternal blood metals and child history of KD.

Quartile of metal level (ng/g)	KD cases (N = 316)	Non-KD cases (N = 85,062)	Crude model		Adjusted model^a^	
			OR (95% CI)	*p* value	Adjusted OR (95% CI)	*p* value
Hg						
Q1 (< 2.56)	73	21,273	1.00 (referent)		1.0 (referent)	
Q2 (2.57–3.65)	87	21,407	1.18 (0.86–1.61)	0.287	1.13 (0.71–1.80)	0.599
Q3 (3.66–5.20)	74	21,123	1.02 (0.73–1.41)	0.900	1.21 (0.77–1.93)	0.397
Q4 (> 5.21)	82	21,259	1.12 (0.81–1.54)	0.468	1.29 (0.82–2.03)	0.263
			P for trend = 0.677		P for trend = 0.373	
Cd						
Q1 (< 0.495)	71	21,367	1.00 (referent)		1.0 (referent)	
Q2 (0.496–0.660)	80	21,204	1.13 (0.82–1.56)	0.437	0.93 (0.59–1.49)	0.785
Q3 (0.661–0.899)	88	21,240	1.24 (0.91–1.70)	0.167	1.33 (0.87–2.04)	0.181
Q4 (> 0.900)	77	21,251	1.09 (0.78–1.50)	0.599	0.99 (0.63–1.58)	0.999
			P for trend = 0.575		P for trend = 0.258	
Pb						
Q1 (< 4.69)	75	21,336	1.00 (referent)		1.0 (referent)	
Q2 (4.70–5.84)	92	21,336	1.22 (0.90–1.66)	0.190	1.29 (0.85–1.96)	0.223
Q3 (5.85–7.31)	83	21,130	1.11 (0.81–1.52)	0.487	0.90 (0.57–1.42)	0.667
Q4 (> 7.31)	66	21,260	0.88 (0.63–1.23)	0.462	0.84 (0.52–1.34)	0.468
			P for trend = 0.197		P for trend = 0.201	
Se						
Q1 (< 156)	69	21,341	1.00 (referent)		1.0 (referent)	
Q2 (157–168)	83	21,714	1.18 (0.85–1.62)	0.305	1.91 (1.21–3.02)	0.005
Q3 (169–182)	87	21,879	1.22 (0.89–1.68)	0.200	1.36 (0.84–2.21)	0.206
Q4 (> 183)	77	20,128	1.18 (0.85–1.63)	0.311	1.17 (0.70–1.94)	0.542
			P for trend = 0.590		P for trend = 0.056	
Mn						
Q1 (< 12.6)	75	21,656	1.00 (referent)		1.0 (referent)	
Q2 (12.6–15.3)	89	21,053	1.22 (0.89–1.66)	0.204	1.01 (0.66–1.54)	0.935
Q3 (15.4–18.6)	79	21,290	1.07 (0.78–1.47)	0.669	0.90 (0.59–1.39)	0.661
Q4 (> 18.7)	73	21,063	1.00 (0.72–1.38)	0.996	0.70 (0.44–1.11)	0.138
			P for trend = 0.541		P for trend = 0.270	

^a^Adjusted with Covariates, presence or absence of siblings, infant feeding, mother's history of use of medications during pregnancy, mother's history of allergy, child's history of common cold, child's history of gastroenteritis, child's history of allergy, and socioeconomic factors.

Three sensitivity analyses were performed: first for the extension of the outcome to C3Y and second for the conversion to the ordinary logarithm. The conversion to a normal logarithm was performed as shown in Figure S1, with each heavy metal concentration normally distributed by conversion to the natural logarithm. The ORs were then calculated as shown in Table S1. There was no increase in the ORs with increasing concentrations of heavy metals. Tables S2 and S3 show the extended outcome of KD onset at C2Y and C3Y. Table S4 shows the results of the analysis as continuous variables. These were no increase in the odds ratio associated with concentration, and no clear significant difference.

## Discussion

In this study, we used a large birth cohort study of approximately 100,000 mother–child pairs, which showed no significant association between maternal blood heavy metal concentrations during pregnancy and KD incidence at C1Y. Moreover, there were no significant differences between the number of cases of high and low maternal concentrations of mercury, cadmium, lead, selenium, and manganese during pregnancy and the ORs for KD incidence at C1Y. We also performed two sensitivity analyses to improve the robustness of the results: first, the log transformation included a normalization of the right hem distribution, which allowed for a more detailed examination of the causal relationship between maternal blood heavy metal levels and KD incidence; second, the extended outcome included C2Y and C3Y, which are the second and third most common ages of KD onset.

A comparison of each heavy metal concentration with previous reports is described. Mercury concentrations were comparable to those in the blood of pregnant women in Japan in 2006–2007 and Taiwan in 2010–11^10,11^. The ratio is almost ten times higher than that of pregnant women in the US^12^, which may be attributed to a high seafood intake. Cadmium has been decreased by one-tenth compared to the 1984 data in Japan^13^, which is similar to other developed countries, with rice as the presumed primary source^14^. Lead showed a similar change as cadmium^12,13^. Selenium and manganese were similar to studies in China, Korea, the UK, and Canada^9,15–17^.

Heavy metal exposure is an important epidemiological health issue. The National Institute of Environmental Health Sciences has declared combined metal and chemical exposure in life, which depends on living environment and dietary habits, as a priority research area^18^. Mercury, cadmium, and lead, which cause various health problems^19^, are transmitted from the mother to the fetus^20^ and increase the risk of disruption of fetal development, including preterm birth^21^, nervous system abnormalities^22^, and impaired mental function^23^. The association between heavy metal exposure and KD was previously indicated after the KD was first reported, and the controversy continues to this date.

Mercury is also a major discussed issue. Generally, the main health effect of methylmercury is neurotoxicity due to exposure to high concentrations which became known to the world in 1956 in the Kyushu region of Japan as Minamata disease^24^. In recent years, exposure to high concentrations has been eliminated in developed countries, but there are still effects of exposure in the low-concentration range. For instance, it has been shown that fetal methylmercury exposure is associated with decreased verbal comprehension and learning, and it is necessary to examine various physical effects even at low concentrations. In this study, we focused on the similarities between the symptoms of acrodynia, a mercury poisoning, and KD. Acrodynia is methylmercury poisoning in children, associated with peripheral skin erythema, mouth erythema, and skin rash. For mercury and KD, almost all previous reports are short case reports, and several are prospective studies. Orlowski et al. used 24-h urine samples of six patients with KD and compared their mercury levels to those of six non-KD patients with similar characteristics who were hospitalized at the same time. Patients with KD had abnormally high urinary mercury excretion^25^. In contrast, Chang et al. measured blood mercury levels in 85 patients with KD from 2016 to 2020 and examined the association between treatment resistance and coronary artery aneurysms. The results showed no significant differences or association between blood mercury levels and KD outcomes^26^.

One mercury concentration-dependent factor is dietary habits, especially seafood^27^. In Asian countries along the seacoast, such as Japan and Taiwan, national blood mercury levels are higher than in Western countries^28^. The reasons for this have been suggested to be the consumption of seafood as the primary source of mercury in addition to other factors, such as industrial emissions to the soil surface, age, and race. In our study, maternal blood mercury levels were higher than those reported in Europe and US^29,30^. Although blood heavy metals are transmitted to the fetus through the placenta, we found that this transmission does not increase the risk of KD onset. In other words, our results suggest that daily seafood intake does not increase KD incidence. According to a national survey of KD in Japan, the incidence of KD has increased from 88 per 100,000 in 1990, 141 in 2000, 242 in 2010, and 370 in 2019^31^. In contrast, the annual mercury intake of the Japanese population has been decreasing^32^. The data shown are until 2000, but the dietary habit of favoring meat over fish has stayed the same. It is expected to remain unchanged and not increase significantly in recent years^33^. Therefore, KD is increasing, but mercury intake has remained constant or decreased, indicating that mercury may not significantly cause Kawasaki disease.

Another hypothesis for the metabolic regulation of mercury is ethnic differences. Genome-wide association studies have identified several genetic loci as susceptibility factors for KD, including the inositol 1,4,5-triphosphate kinase C (*ITPKC)* gene, which encodes the ITPKC protein involved in regulatory T cell activation^34^. SNPs in *ITPKC* were associated with KD incidence and treatment resistance^35^. However, the present study was conducted in a Japanese population, and no data on genetic variation were collected; therefore, it was difficult to determine these racial differences and genetic variation.

The strength of this study is that it is the first report of an association between maternal blood heavy metals, including mercury, and KD incidence in a large population cohort. We believe that our study reflects the general population because it is similar to the proportion of KD and the distribution of maternal blood heavy metal concentrations. According to the Japanese annual surveillance of KD, the prevalence has been reported as 330 per 100,000 individuals^3,36^. The distribution of blood heavy metal concentrations in pregnant women also corresponded to that reported previously^14^.

The study had several limitations. First, incidence of KD in JECS was based on questionnaires to mothers every 6 months. There is a possibility of self-report bias, and we were unable to obtain specific onset dates for each case or detailed clinical information such as blood tests. Second, the measured blood heavy metal concentrations were those of the mothers. However, as noted before, maternal heavy metals are transferred to the fetus, and infants are mainly fed on breast milk or artificial milk, with little variety in oral intake, so we consider causality to be sufficiently probative. Third, there is the possibility of residual confounding factors. For example, acute infection with a specific virus or a detailed family history were not included in the JECS database. Fourth, the JECS database included only Japanese children, which may have limited the external validity of our findings. Fifth, co-exposure was not taken into account, and the association of variables combined was not examined, however, this data will serve as a basis for similar studies in the future.

Despite these limitations, there have been no previous reports examining the association of heavy metals in blood with KD of this scale, and we think it will contribute to the debate over the cause of KD. We conclude that the likelihood of heavy metals in blood as a cause of KD is limited, and indirectly, the association between oral intake and KD in children is also considered to be less plausible.

Moreover, the health effects of heavy metal exposure will continue to be a matter that needs to be closely monitored as society develops. JECS is an ongoing birth cohort, and participants are continuously being followed. There have been reported associations between heavy metals in maternal blood and low birth weight^37^, neurodevelopmental disorders in infants^38^, isolated cleft lip and palate^39^, etc. Further data are accumulated, and more associations with more diseases may be discovered.

## Conclusion

In this study, we found a non-significant association between maternal blood heavy metal concentrations during pregnancy and KD incidence in children. There has been no study with a large birth cohort on a similar topic, and this study provides an implication for the long-debated etiology of KD. Our study suggests that daily dietary intake of seafood may not increase the risk of KD onset.

## Methods

### Database

This study used the JECS database, an ongoing nationwide prospective birth cohort study in Japan. It was initially designed to study the association between environmental factors and maternal and child health from prenatal period to adulthood^40,41^. The JECS consists of Regional Centres across Japan (Hokkaido, Miyagi, Fukushima, Chiba, Kanagawa, the Koshin region, Toyama, Aichi, Kyoto, Osaka, Hyogo, Tottori, Kochi, Fukuoka, and South Kyushu/Okinawa). Recruitment began in January 2011 and ended in March 2014 at participating Co-operating health care providers and local government office. Approximately, 100,000 pregnant women participated. A dedicated staff member explained the study in face-to-face encounters and obtained informed consent from all pregnant women to participate in the study. The schedule of medical examinations is presented in Reference^42,43^.

In this study, we used the data from a dataset jecs-ta-20190930 of pregnant women in the first (MT1) and second and third (MT2) trimesters, medical record transcriptions of newborns at birth (Dr0m), and questionnaires answered by the mothers of their babies at age 1 (C1Y), 2 (C2Y), and 3 (C3Y) years. We analyzed maternal and familial disease history, maternal blood heavy metal levels in MT2, abnormalities in Dr0m (e.g., neonatal asphyxia), KD occurrence in children during C1Y–C3Y, and infectious or allergic disease occurrences in C1Y.

### Participants, exposure, outcome, covariates

Eligible participants were mother–child pairs with singleton pregnancies who had data on blood heavy metal concentrations and a written questionnaire at C1Y. Multiple pregnancies were excluded to avoid different outcomes from a singular maternal exposure.

The exposures were the blood levels of five heavy metals, methyl-mercury (mercury), cadmium, lead, selenium, and manganese are measured in MT2, the JECS cohort study focuses on these five heavy metals due to their health impacts^14^. Mercury, cadmium, and lead are known to harm children's health and development, while selenium and manganses, although essential, can be neurotoxic or toxic at high levels^44–46^. For more details on the specimen collection methods, measurement, and quality control, see reference^14^. Regarding KD, mercury is a primary concern for its symptom similarity to mercury poisoning^4–6^, and its link with KD has been reported but remains unresolved. Cadmium is also associated with KD^47^, whereas lead, selenium, and manganese lack clear links. However, their harmful effects and the novelty of studying them in a large-scale context like JECS warrant their inclusion.

The outcome is the KD incidence below age C1Y, based on the questionnaires answered by the mothers. The outcome at C1Y is based on the hypothesis that the remaining effects of prenatal exposure are likely to persist for approximately one year. For the sensitivity analysis, the KD incidence at age C2Y and C3Y was also evaluated.

The covariates are all considered to be associated with the incidence of KD based on previous reports. The presence or absence of a sibling^48^, the common cold four or more times per year^49^, and a history of allergies^50,51^ were incorporated as factors influencing the immune system response and development. Feeding method, complete breastfeeding, mixed, or artificial nutrition, is a factor that influences nutritional status^52^. Breastfeeding was included because previous studies have shown that breastfeeding significantly reduces the odds ratio for hospitalization. Maternal infections and allergies during pregnancy^53^ were included as factors affecting fetal development. Socioeconomic factors and gestational week, gender, and birth weight were included as basic characteristics in epidemiological studies that may substantially influence health at birth^54,55^.

### Statistical analysis and ethics approval

According to the KD incidence, we compared the groups for each blood heavy metal concentration and covariate using the Mann–Whitney U test. Next, maternal blood heavy metal concentrations were classified into quartiles (Q1–Q4)^56^. This is because the heavy metal concentrations were not normally distributed and we wanted to reduce the influence of outliers in the data. The distribution of heavy metal concentrations was skewed to the left overall as shown in Figure S1, and outliers with abnormally high values were also present. An additional reason was the lack of a clear threshold for disease incidence associated with each heavy metal concentration, which made it difficult to set arbitrary cutoff values; therefore, quartiles were used. Finally, the odds ratios (OR) for Q2–Q4 were calculated by multivariate logistic regression using the lowest concentration quartile (Q1) as a reference. A sensitivity analysis was performed using three methods. First, as the heavy metal concentrations were not normally distributed, they were converted to normal logarithms and then divided into quartiles, and multivariate logistic regression analysis was carried out. Second, we extended the outcome using the data on KD onset in C2Y and C3Y. Third, heavy metal concentrations were analyzed as continuous variables. The tests were all two-tailed, and a p-value < 0.05 was considered statistically significant. All statistical analyses were carried out using Stata/SE Version 17.0 (Stata Corp, US).

The JECS protocol was reviewed and approved by the Ministry of the Environment’s Institutional Review Board on Epidemiological Studies and the Ethics Committees of all participating institutions [Ethical Number 100910001]. This study was performed in accordance with the Declaration of Helsinki.

### Supplementary Information

Supplementary Information.

## Data Availability

Data are unsuitable for public deposition due to ethical restrictions and the legal framework of Japan. It is prohibited by the Act on the Protection of Personal Information (Act No. 57 of 30 May 2003, amendment on 9 September 2015) to publicly deposit data containing personal information. Ethical Guidelines for Medical and Health Research Involving Human Subjects enforced by the Japan Ministry of Education, Culture, Sports, Science and Technology and the Ministry of Health, Labour and Welfare also restricts the open sharing of epidemiologic data. All inquiries about access to data should be sent to jecs-en@nies.go.jp. The person responsible for handling inquiries sent to this e-mail address is Dr Shoji F. Nakayama, JECS Programme Office, National Institute for Environmental Studies.

## Abbreviations

KD: Kawasaki Disease
JECS: Japan Environment and Children's Study
Hg: Mercury
Cd: Cadmium
Pb: Lead
Se: Selenium
Mn: Manganese
CI: Confidence interval
OR: Odds ratio
ITPKC: Inositol 1,4,5-triphosphate kinase C
