# Supplementary material for: Association between maternal heavy metal exposure and Kawasaki Disease, the Japan Environment and Children’s Study (JECS)
Source: Sci Rep. 2024 Apr 30;14:9947. doi: 10.1038/s41598-024-60830-z (PMC11061304; doi:10.1038/s41598-024-60830-z)
Supplement: Supplementary file 1 — Supplementary Information. [file 41598_2024_60830_MOESM1_ESM.docx]

**Supplymentary tables and figures**

Title: Associations between maternal heavy metal exposure and Kawasaki Disease: using the Japan Environment and Children’s Study (JECS) cohort

**Figure S1.** Sensitivity analysis, histograms of maternal blood heavy metal concentrations converted to normal logarithms.

|  | Raw value | Logarithm (base 10) |
| --- | --- | --- |
| Hg | 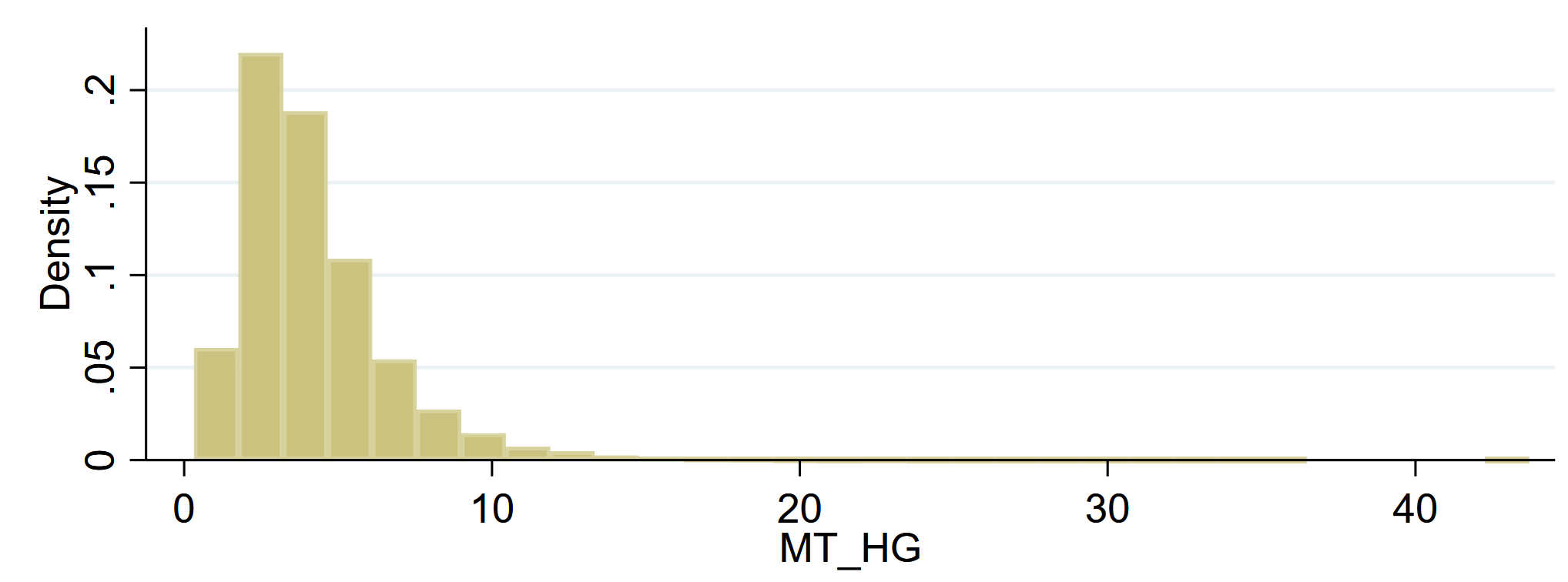 | 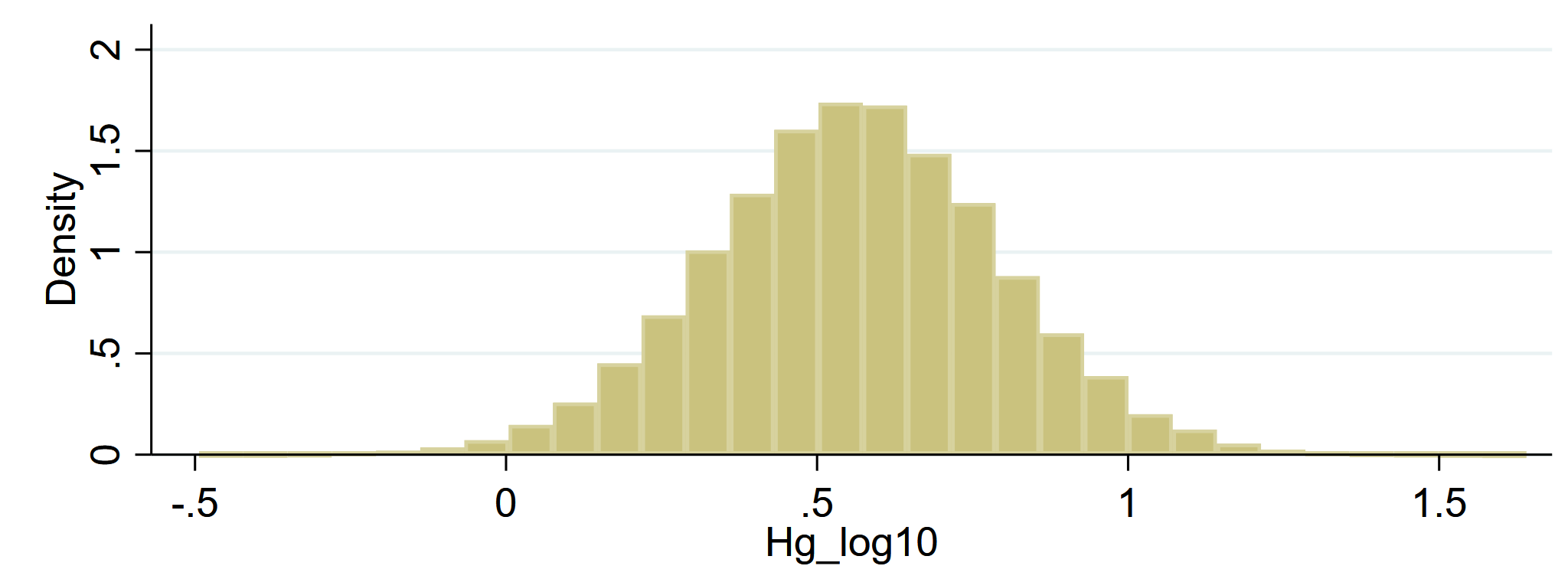 |
| Cd | 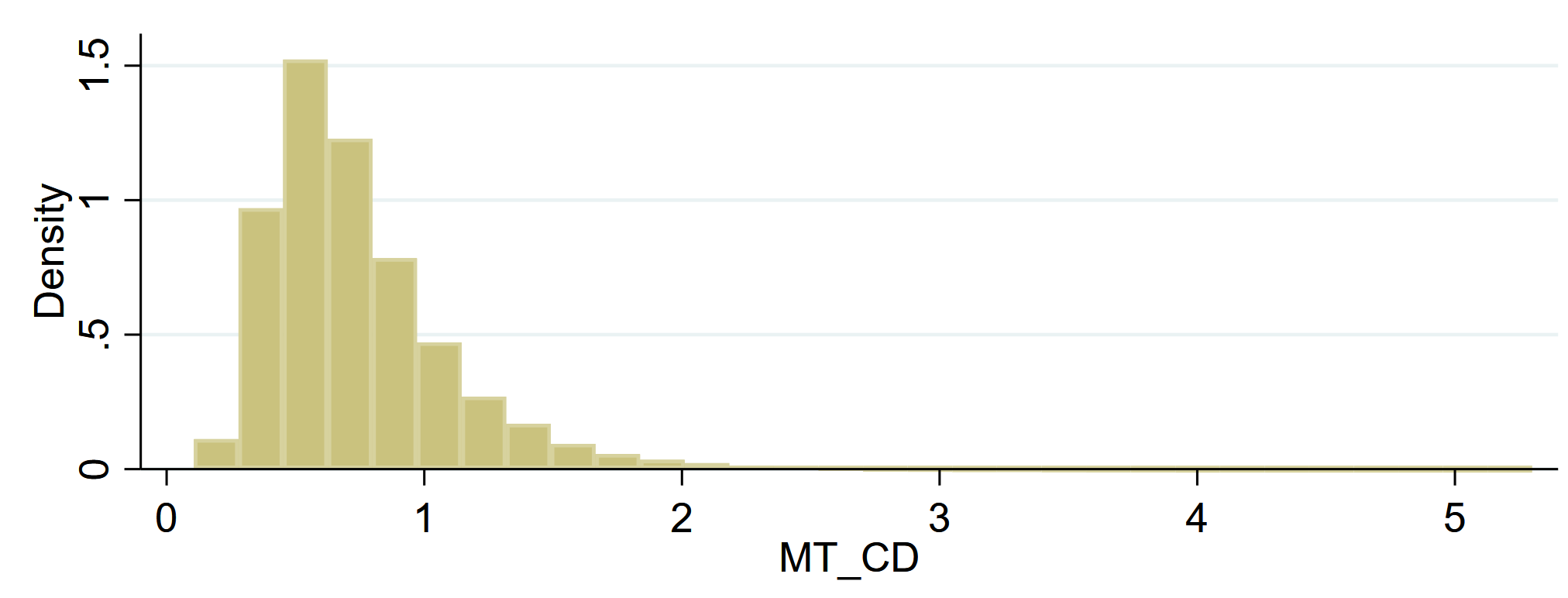 | 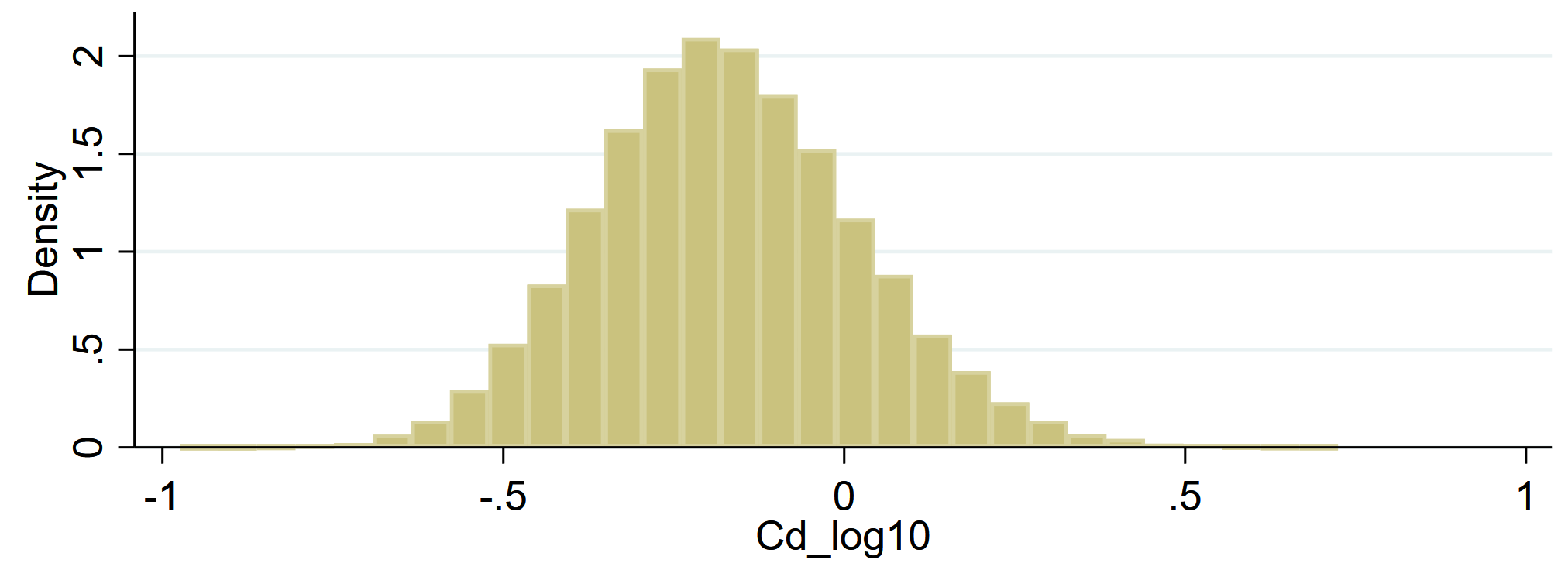 |
| Pb | 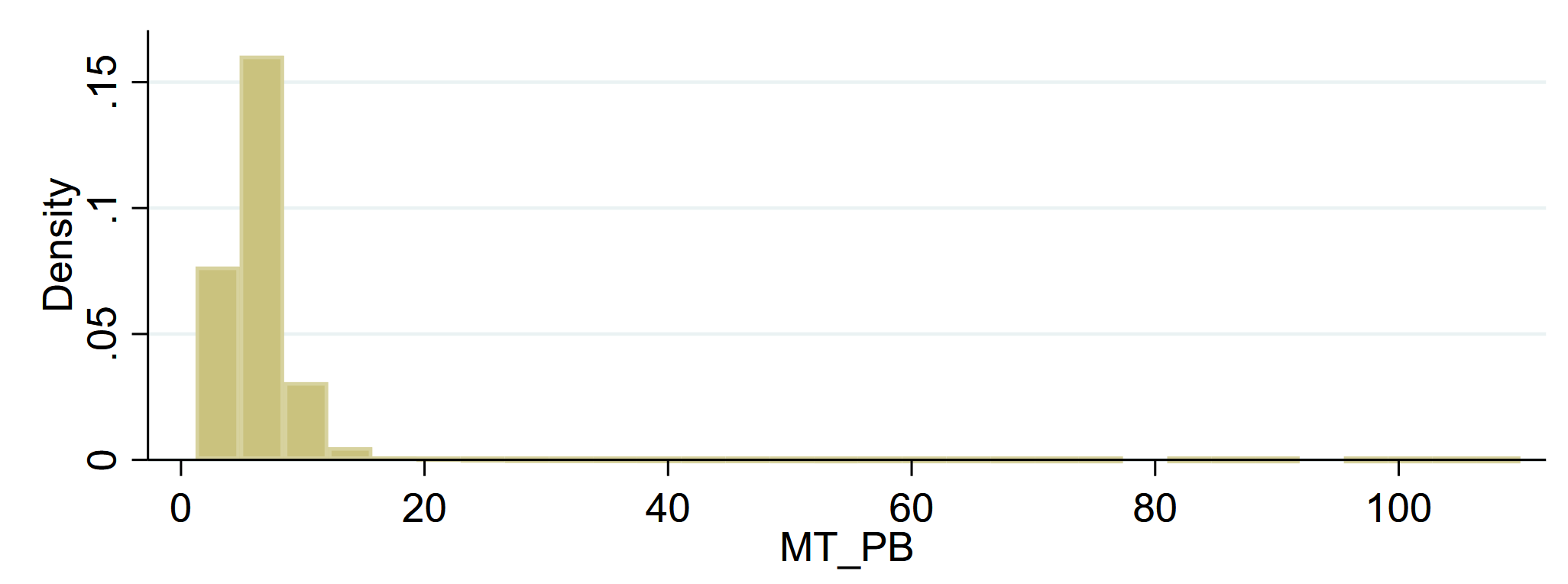 | 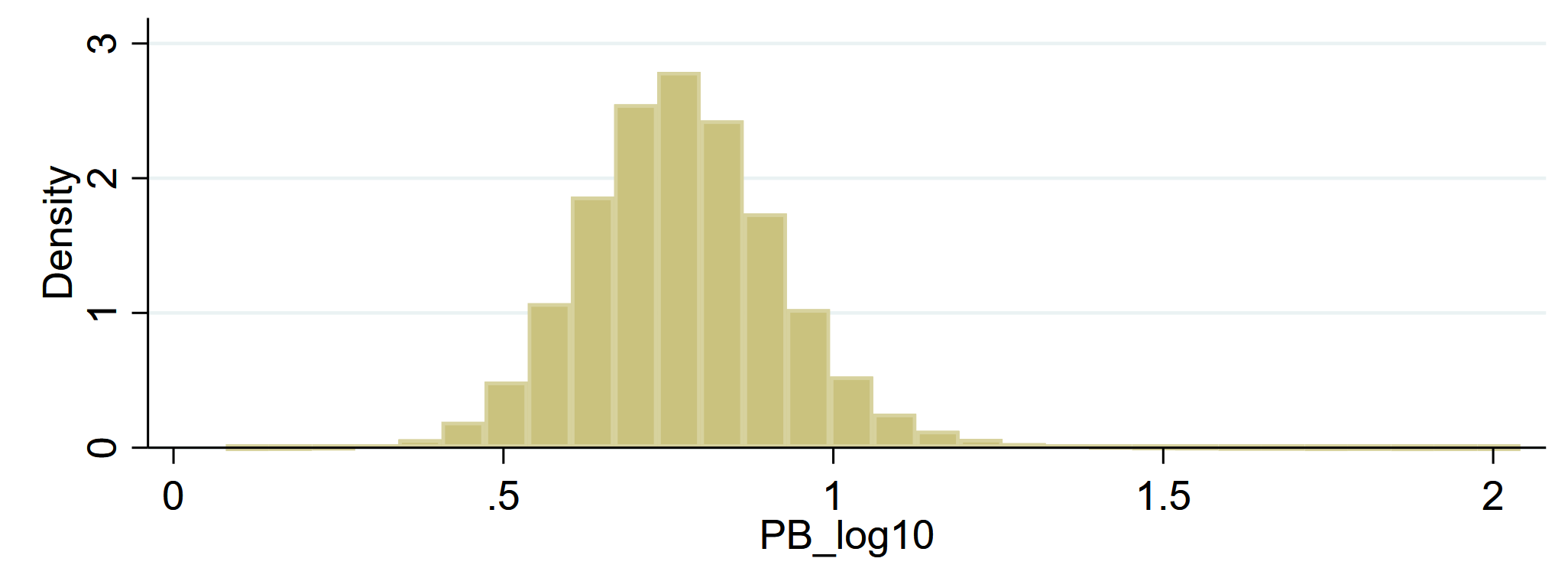 |
| Se | 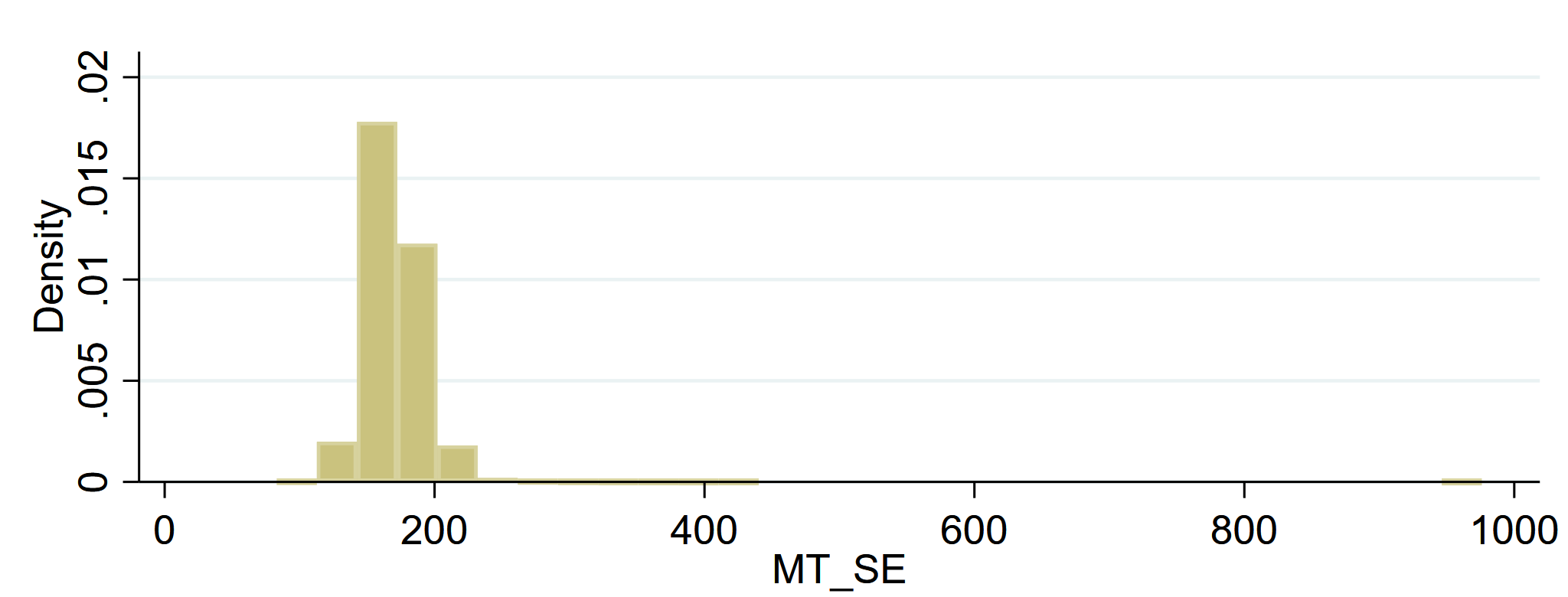 | 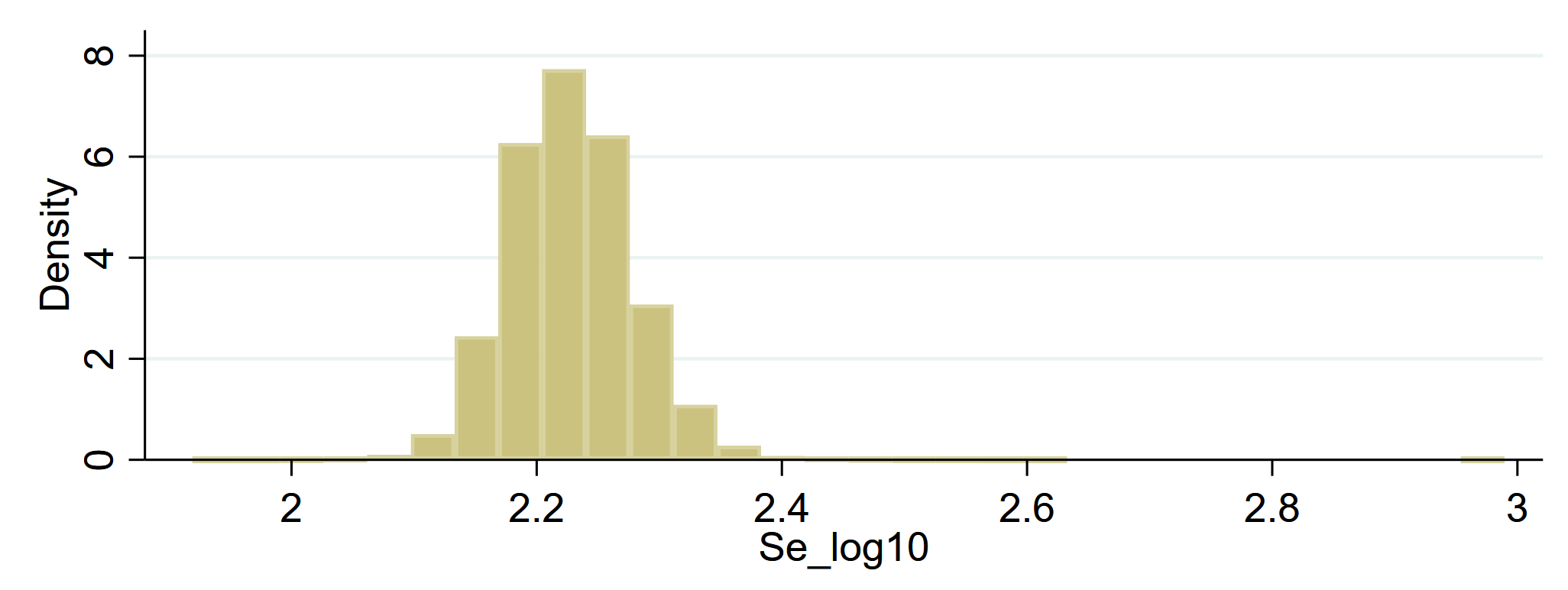 |
| Mn | 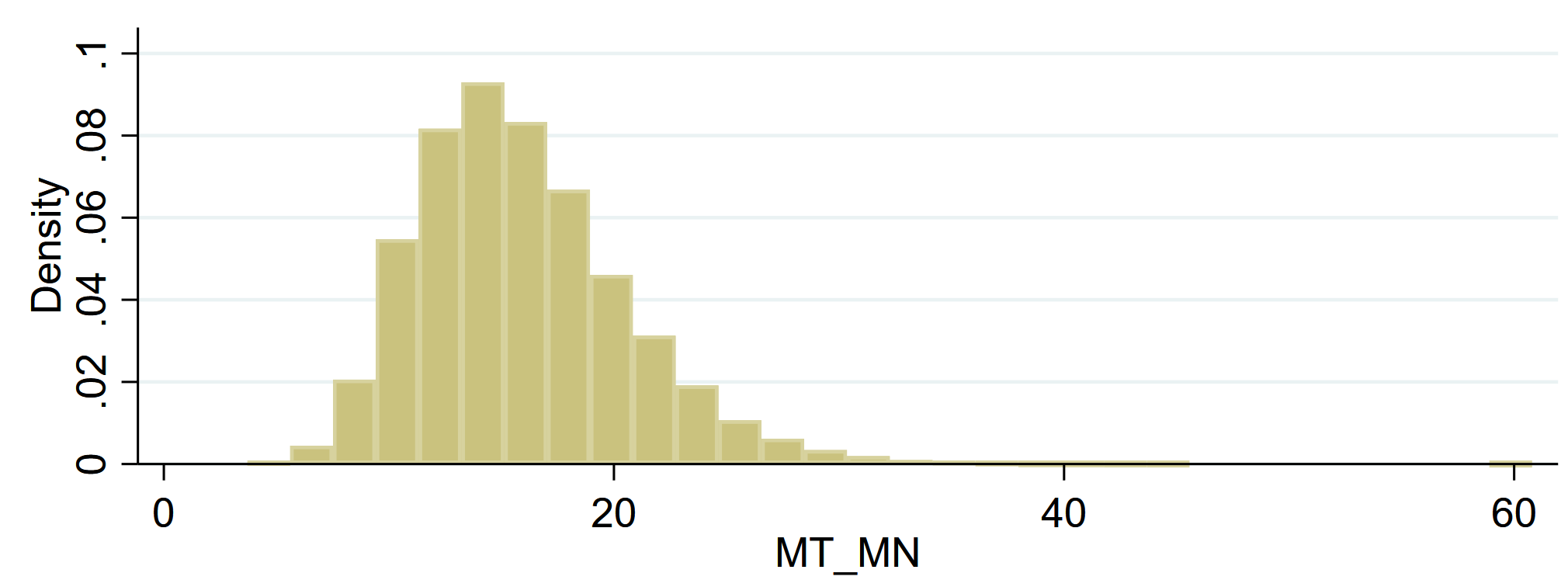 | 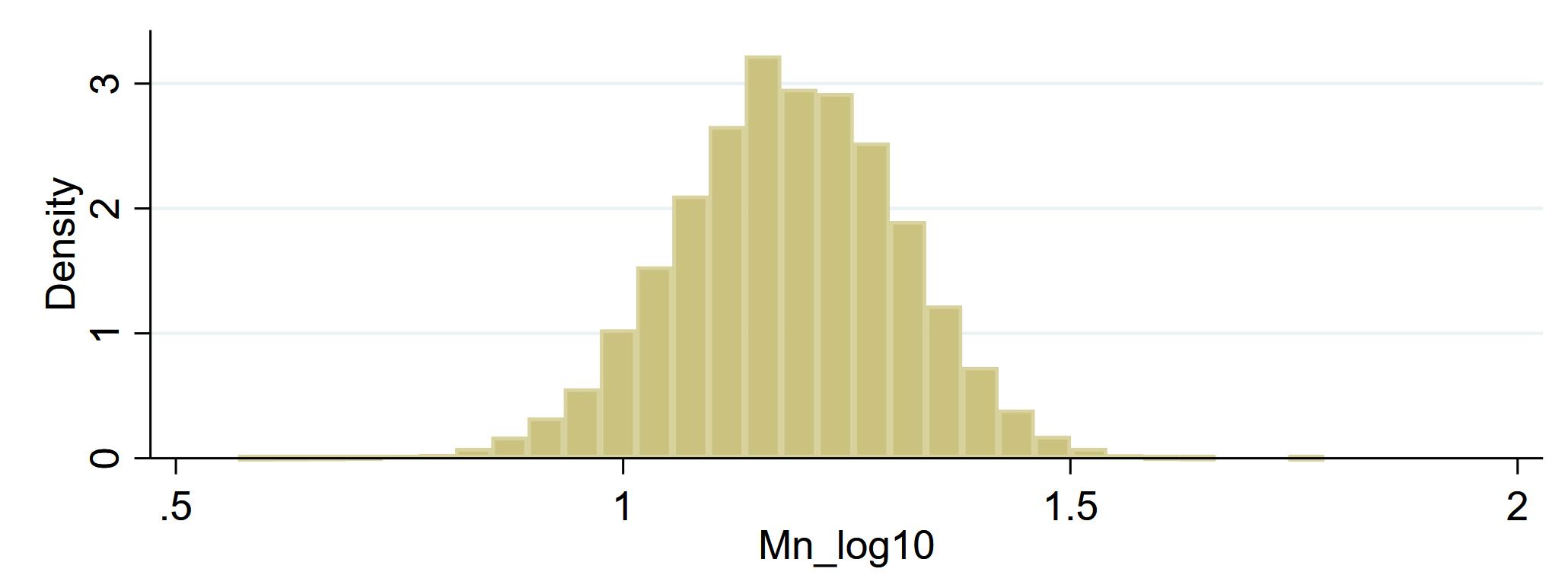 |

**Table S1.** Sensitivity analysis, converted to normal logarithms.

| Quartile of metal level (ng/g) | Model s1: normal logarithms | | | |
| --- | --- | --- | --- | --- |
|  | KD cases  (n=316 | Non-KD cases  (n=85062) | Adjusted OR (95%CI)^a^ | p value |
| Hg |  |  |  |  |
| Q1 | 73 | 21273 | 1.0 (referent) |  |
| Q2 | 87 | 21407 | 1.13 (0.71-1.80) | 0.599 |
| Q3 | 74 | 21123 | 1.21 (0.77-1.93) | 0.397 |
| Q4 | 82 | 21259 | 1.29 (0.82-2.03) | 0.263 |
|  |  |  | P for trend = 0.373 |  |
| Cd |  |  |  |  |
| Q1 | 71 | 21367 | 1.0 (referent) |  |
| Q2 | 80 | 21204 | 0.93 (0.59-1.49) | 0.785 |
| Q3 | 88 | 21240 | 1.33 (0.87-2.04) | 0.181 |
| Q4 | 77 | 21251 | 0.99 (0.63-1.58) | 0.999 |
|  |  |  | P for trend = 0.258 |  |
| Pb |  |  |  |  |
| Q1 | 75 | 21336 | 1.0 (referent) |  |
| Q2 | 92 | 21336 | 1.29 (0.85-1.96) | 0.223 |
| Q3 | 83 | 21130 | 0.90 (0.57-1.42) | 0.667 |
| Q4 | 66 | 21260 | 0.84 (0.52-1.34) | 0.468 |
|  |  |  | P for trend = 0.201 |  |
| Se |  |  |  |  |
| Q1 | 69 | 21341 | 1.0 (referent) |  |
| Q2 | 83 | 21714 | 1.91 (1.21-3.02) | 0.005 |
| Q3 | 87 | 21879 | 1.36 (0.84-2.21) | 0.206 |
| Q4 | 77 | 20128 | 1.17 (0.70-1.94) | 0.542 |
|  |  |  | P for trend = 0.056 |  |
| Mn |  |  |  |  |
| Q1 | 75 | 21656 | 1.0 (referent) |  |
| Q2 | 89 | 21053 | 1.01 (0.66-1.54) | 0.935 |
| Q3 | 79 | 21290 | 0.90 (0.59-1.39) | 0.661 |
| Q4 | 73 | 21063 | 0.70 (0.44-1.11) | 0.138 |
|  |  |  | P for trend = 0.270 |  |

^a^ Adjusted with Covariates, presence or absence of siblings, infant feeding, mother's history of use of medications during pregnancy, mother's history of allergy, child's history of common cold, child's history of gastroenteritis, child's history of allergy, and socioeconomic factors.

**Table S2.** Sensitivity analysis, extended to the outcome to 2-year-old age.

| Quartile of metal level (ng/g) | Model s2: outcome to 2-year-old age | | | |
| --- | --- | --- | --- | --- |
|  | KD cases  (n=620) | Non-KD cases  (n=78993) | Adjusted OR (95%CI)^a^ | p value |
| Hg |  |  |  |  |
| Q1 (<2.56) | 157 | 19639 | 1.00 (referent) |  |
| Q2 (2.57-3.65) | 175 | 19814 | 0.94 (0.69-1.28) | 0.709 |
| Q3 (3.66-5.20) | 137 | 19707 | 0.90 (0.66-1.23) | 0.528 |
| Q4 (>5.21) | 151 | 19833 | 1.02 (0.75-1.38 | 0.886 |
|  |  |  | P for trend = 0.199 |  |
| Cd |  |  |  |  |
| Q1 (<0.495) | 147 | 19859 | 1.00 (referent) |  |
| Q2 (0.496-0.660) | 148 | 19717 | 0.82 (0.60-1.12) | 0.215 |
| Q3 (0.661-0.899) | 170 | 19737 | 1.00 (0.74-1.34) | 0.984 |
| Q4 (>0.900) | 155 | 19680 | 0.91 (0.67-1.24) | 0.580 |
|  |  |  | P for trend = 0.146 |  |
| Pb |  |  |  |  |
| Q1 (<4.69) | 148 | 19846 | 1.00 (referent) |  |
| Q2 (4.70-5.84) | 172 | 19859 | 1.16 (0.86-1.56) | 0.326 |
| Q3 (5.85-7.31) | 157 | 19616 | 0.99 (0.72-1.35) | 0.977 |
| Q4 (>7.31) | 143 | 19672 | 0.93 (0.68-1.29) | 0.701 |
|  |  |  | P for trend = 0.148 |  |
| Se |  |  |  |  |
| Q1 (<156) | 147 | 19819 | 1.00 (referent) |  |
| Q2 (157-168) | 145 | 20220 | 1.10 (0.80-1.51) | 0.544 |
| Q3 (169-182) | 173 | 20297 | 1.16 (0.85-1.58) | 0.343 |
| Q4 (>183) | 155 | 18657 | 1.11 (0.81-1.53) | 0.501 |
|  |  |  | P for trend = 0.191 |  |
| Mn |  |  |  |  |
| Q1 (<12.6) | 176 | 20141 | 1.00 (referent) |  |
| Q2 (12.6-15.3) | 150 | 19608 | 0.85 (0.63-1.14) | 0.283 |
| Q3 (15.4-18.6) | 146 | 19735 | 0.75 (0.55-1.01) | 0.064 |
| Q4 (>18.7) | 148 | 19509 | 0.75 (0.55-1.02) | 0.075 |
|  |  |  | P for trend = 0.796 |  |

^a^ Adjusted with Covariates, presence or absence of siblings, infant feeding, mother's history of use of medications during pregnancy, mother's history of allergy, child's history of common cold more than four times a year, child's history of allergy, and socioeconomic factors.

**Table S3.** Sensitivity analysis, extended to the outcome to 3-year-old age.

| Quartile of metal level (ng/g) | Model s3: outcome to 3-year-old age | | | |
| --- | --- | --- | --- | --- |
|  | KD cases  (n=839) | Non-KD cases  (n=74307) | Adjusted OR (95%CI)^a^ | p value |
| Hg |  |  |  |  |
| Q1 (<2.56) | 96 | 18820 | 1.00 (referent) |  |
| Q2 (2.57-3.65) | 113 | 19011 | 1.12 (0.78-1.62) | 0.518 |
| Q3 (3.66-5.20) | 108 | 18934 | 1.12 (0.78-1.63) | 0.519 |
| Q4 (>5.21) | 79 | 18993 | 0.69 (0.45-1.05) | 0.085 |
|  |  |  | P for trend = 0.212 |  |
| Cd |  |  |  |  |
| Q1 (<0.495) | 90 | 19002 | 1.00 (referent) |  |
| Q2 (0.496-0.660) | 98 | 18914 | 1.04 (0.70-1.53) | 0.832 |
| Q3 (0.661-0.899) | 115 | 18958 | 1.40 (0.97-2.02) | 0.066 |
| Q4 (>0.900) | 93 | 18884 | 0.94 (0.62-1.41) | 0.772 |
|  |  |  | P for trend = 0.316 |  |
| Pb |  |  |  |  |
| Q1 (<4.69) | 106 | 19068 | 1.00 (referent) |  |
| Q2 (4.70-5.84) | 91 | 19031 | 0.91 (0.62-1.32) | 0.624 |
| Q3 (5.85-7.31) | 88 | 18775 | 0.75 (0.50-1.11) | 0.157 |
| Q4 (>7.31) | 111 | 18884 | 1.10 (0.77-1.57) | 0.590 |
|  |  |  | P for trend = 0.421 |  |
| Se |  |  |  |  |
| Q1 (<156) | 101 | 19057 | 1.00 (referent) |  |
| Q2 (157-168) | 116 | 19378 | 1.14 (0.79-1.65) | 0.468 |
| Q3 (169-182) | 106 | 19468 | 1.20 (0.83-1.73) | 0.316 |
| Q4 (>183) | 73 | 17855 | 0.74 (0.49-1.14) | 0.179 |
|  |  |  | P for trend = 0.270 |  |
| Mn |  |  |  |  |
| Q1 (<12.6) | 102 | 19375 | 1.00 (referent) |  |
| Q2 (12.6-15.3) | 107 | 18811 | 1.28 (0.87-1.89) | 0.196 |
| Q3 (15.4-18.6) | 82 | 18878 | 1.23 (0.83-1.82) | 0.289 |
| Q4 (>18.7) | 105 | 18694 | 1.25 (0.84-1.85) | 0.261 |
|  |  |  | P for trend = 0.575 |  |

^a^ Adjusted with Covariates, presence or absence of siblings, infant feeding, mother's history of use of medications during pregnancy, mother's history of allergy, child's history of common cold more than four times a year, child's history of allergy, and socioeconomic factors.

**Table S4.** Sensitivity analysis, analyzed as a continuous variable.

| Metal | Model s4: continuous variable | | | |
| --- | --- | --- | --- | --- |
|  | KD cases (n=316) | Non-KD cases (n=85062) | Adjusted OR (95%CI)^a^ | p value |
| Hg | (same as above) | | 1.02 (0.96-1.08) | 0.395 |
| Cd |  |  | 1.10 (0.72-1.66) | 0.646 |
| Pb |  |  | 0.97 (0.91-1.04) | 0.450 |
| Se |  |  | 0.99 (0.99-1.01) | 0.638 |
| Mn |  |  | 0.96 (0.93-1.00) | 0.075 |

^a^ Adjusted with Covariates, presence or absence of siblings, infant feeding, mother's history of use of medications during pregnancy, mother's history of allergy, child's history of common cold, child's history of gastroenteritis, child's history of allergy, and socioeconomic factors.
